# Cerebellar contributions to dystonia: unraveling the role of Purkinje cells and cerebellar nuclei

**DOI:** 10.3389/dyst.2025.14006

**Published:** 2025-02-16

**Authors:** Nichelle N. Jackson, Jacob A. Stagray, Heather D. Snell

**Affiliations:** 1 Department of Neuroscience, Yale School of Medicine, New Haven, CT, United States,; 2 Wu Tsai Institute, Yale School of Medicine, New Haven, CT, United States

**Keywords:** cerebellum, Purkinje cells, cerebellar nuclei, dystonia, calcium handling

## Abstract

Dystonias are a group of neurodegenerative disorders that result in altered physiology associated with motor movements. Both the basal ganglia and the cerebellum, brain regions involved in motor learning, sensory perception integration, and reward, have been implicated in the pathology of dystonia, but the cellular and subcellular mechanisms remain diverse and for some forms of dystonia, elusive. The goal of the current review is to summarize recent evidence of cerebellar involvement in different subtypes of dystonia with a focus on Purkinje cell (PC) and cerebellar nuclei (CN) dysfunction, to find commonalities in the pathology that could lay the groundwork for the future development of therapeutics for patients with dystonia. Here we will briefly discuss the physical and functional connections between the basal ganglia and the cerebellum and how these connections could contribute to dystonic symptoms. We proceed to use human and animal model data to discuss the contributions of cerebellar cell types to specific dystonias and movement disorders where dystonia is a secondary symptom. Ultimately, we suggest PC and CN irregularity could be a locus for dystonia through impaired calcium dynamics.

## Introduction

The term “dystonia musculorum deformans,” now simply dystonia, was first coined by Oppenheim in 1911 to describe his phenotypic observations about four patients suffering from alternating muscular hypotonia and hypertonia [1, 2]. Most recently, dystonia has been defined as a class of movement disorders characterized by either sustained or intermittent abnormal muscle contractions. These dystonic contractions often lead to repetitive movements and/or postures that are typically patterned, twisting, and potentially tremulous in nature [3]. Furthermore, some dystonias are worsened by the initiation of voluntary actions associated with increased muscle activation. The unintentional contraction of muscles is associated with but uniquely distinct from primary dystonic movements.

Due to these symptoms, dystonia was thought to be a disorder of the basal ganglia, which is essential for initiating and controlling voluntary actions. However, recent studies have shown the cerebellum, which is essential for motor learning and coordination, is also involved in the pathogenesis of dystonia. In fact, the cerebellum is an essential region in the motor circuit that includes the basal ganglia, striatum, cortex, and thalamus [4–8]. Human imaging studies characterizing various forms of dystonia have demonstrated multiple regions in this circuit are affected, including the basal ganglia, cortex, cerebellum, and thalamus [7, 9, 10]. This has led researchers to believe dystonia is likely the result of dysfunction along the cortico-striato-thalamo-cortical and/or the cortico-cerebello-thalamo-cortical pathways [8, 11–14]. Is there a locus in these pathways that is common among different dystonias that could be driving the dysfunction in other brain regions irrespective of the etiology? The cerebellum, with its role in sensory integration, motor movement, and extensive connections to cortical regions, could serve as this key locus, particularly due to the pacemaking abilities of Purkinje cells (PC), the main output neuron of the cerebellar cortex. Finding common cerebellar neuronal dysfunction in dystonias could thus lead to a better understanding of underlying mechanisms and set the foundation for the development of targeted therapeutics to help alleviate symptoms in dystonic patients.

Currently, the contribution of the cerebellum to different types of dystonia is not fully understood; however, abnormal PC activity, has been implicated in multiple models of dystonia [15–19]. Additionally, it is well appreciated that cerebellar dysfunction, either in the form of PC or CN irregular firing, underlies many forms of motor impairment [20–22]. The goal of the current review is to summarize recent evidence of cerebellar involvement in different subtypes of dystonia. First, we set the stage by briefly discussing the evolution of the categorization of dystonias. Secondly, we provide a brief overview of the basal ganglia’s involvement in dystonias and discuss how the basal ganglia interacts with the cerebellum. Then, we review the architecture of the cerebellum, which converges onto PCs and CN, and highlight the importance of calcium (Ca^2+^) dynamics in PC intrinsic and synaptic activity. Following this, we review human data and animal models to illustrate the known contributions of each cerebellar cell type to specific dystonias. Finally, we end this review by summarizing known and proposed therapeutic intervention targets. We hypothesize impaired calcium dynamics in PCs and CN lead to firing irregularities that ultimately contribute to the pathogenesis of dystonia. This underlying mechanism of Ca^2+^ dysfunction could contain multiple potential targets for therapeutic interventions.

## Dystonia and the name game

In addition to having well-defined parameters when characterizing dystonia, it is important to ensure specific and consistent nomenclature. This nomenclature should distinguish the unique features of the dystonia subtypes that can consistently be used between patients, clinicians, and researchers. In doing so, we can compare and differentiate the pathological underpinnings of different dystonias, allowing for better therapeutics. Currently, dystonias are classified according to their clinical and etiological features (summarized in Table 1). Clinical features include the age of onset, body distribution, temporal patterning, and association with other movement disorders [3, 23, 24]. An important diagnostic feature is the age of disease onset in patients which can be important for the progression and treatment of dystonia. The distribution of body-associated symptoms is an important feature for both diagnosing patients and providing effective therapeutic treatments. The primary regions of the body affected by dystonia are either focal, segmental, multifocal, generalized, or hemidystonic (Table 1). When characterizing phenotypes associated with generalized dystonia, the disorder can present with or without the involvement of leg movements. The establishment and severity of symptoms can evolve over time. This change is known as temporal patterning for disease pathogenesis. Temporally, dystonias are classified as either static, progressive, persistent, paroxysmal, diurnal, or action-specific. Finally, dystonia can be classified as the sole, or “isolated,” phenotype of the disorder or occur in combination with another neurological disorder affecting movement. An example where dystonia is the sole disorder is DYT-TOR1A (“early-onset generalized dystonia”), while an example of a combination disorder phenotype would be DYT-ATP1A3 (“Rapid-onset dystonia-parkinsonism”).

Etiological features of dystonia include genetic, environmental, or idiopathic causes [3, 23, 24]. Until ~2010s, dystonias associated with a genetic etiology were identified by the prefix “DYT” (for dystonia) and a number between 1 and 34 [23, 25]. More recently, the nomenclature has been updated to identify dystonia with the prefix “DYT” followed by the gene name/locus associated with the disorder. For example, early-onset generalized dystonia, formerly DYT1, is now DYT-TOR1A [25, 26]. Genetic or inherited sources of dystonia can either be autosomal dominant, autosomal recessive, chromosome x-linked, or mitochondrial in origin (Table 1). Environmental factors that contribute to different forms of dystonia can originate through pathology of the nervous system or be acquired from environmental insults like brain injury or drug usage. While dystonia does not appear to have a singular neuropathological origin, certain forms of dystonia are linked to neurodegeneration and/or structural lesions. Dystonia has been found to have other neuropathological causes; however, these forms do not present with degeneration or lesions [3, 23, 24]. Other acquired forms of dystonia are caused by inflammation associated with infections, toxic agents, vascular injuries, neoplasms, or psychological reasons. Finally, there are unknown causes of dystonia that are sporadic or familial in nature.

## Basal ganglia and the cerebellum

While the focus of this review is the cerebellar contribution to dystonia, we would be remiss to leave out a brief discussion of the basal ganglia involvement. Combined and complex dystonias have been historically thought to result from dysfunction of the basal ganglia. In the context of motor control, the basal ganglia ensures correct movements are executed and maintained while inhibiting unwanted movements [27]. However, overactivity of the basal ganglia results in abnormal temporal discrimination in patients with dystonia [28], resulting from delayed responses to a novel environmental stimulus [29]. Abnormal muscle contractions in dystonia patients have also been attributed to an inability to integrate sensory signals in the basal ganglia. In fact, during bouts of dystonic symptoms, many afflicted individuals perform “sensory tricks” which can temporarily alleviate dystonic postures [30, 31]. The basal ganglia however, does not work in isolation, and is connected to, receives inputs from, and sends information to many other brain regions, including the cerebellum. In fact, dystonic muscle contractions have been attributed to abnormal excitability within the motor circuit [32], as well as a decreased connectivity between the premotor cortex, the parietal cortex and basal ganglia.

**Figure 1.**
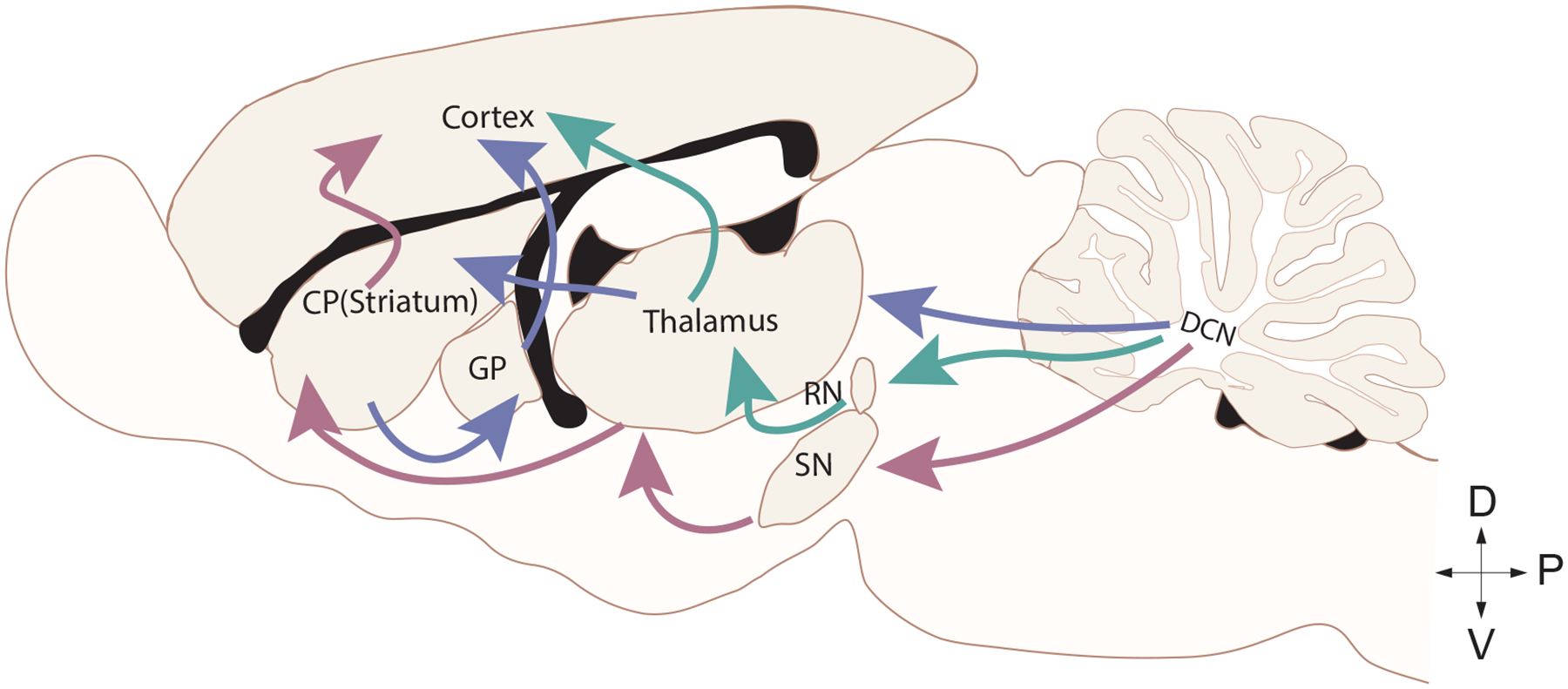
Cerebellar efferent connections to the motor cortex. Sagittal rat brain depicting the direct (red) connection between the cerebellum and the basal ganglia through the substantia nigra (SN), indirect (purple) connection between the cerebellum and the basal ganglia through the thalamus connecting to the corpus striatum (CP) and the globus pallidus (GP), and the canonical (green) cerebellum connection to the motor cortex through the red nucleus (RN) and thalamus.

The functions of the basal ganglia and the cerebellum overlap more than previously thought. Many decades of research have shown the basal ganglia plays a well-established role in choosing between actions and reward-based learning [33, 34], while the cerebellum is involved in motor coordination and motor learning [34, 35]. Recent work, however, shows reward signals in the cerebellum challenging this separation of roles [36–39]. The basal ganglia, which can be separated into multiple components, including the corpus striatum (CP), the subthalamic nucleus (STN), and the substantia nigra (SN; Figure 1), are connected to the cortex and the cerebellum through several interconnected pathways or loops (Figure 1). It’s connections with the cortex are segregated in parallel loops termed “motor” for motor control, “associative” for cognition, and “limbic” for emotional control [40]. The motor loop includes basal ganglia’s connection with the motor cortex through the thalamus.

The basal ganglia and the cerebellum have a direct connection as well as indirect disynaptic connections through the thalamus (Figure 1). Therefore, it is now widely accepted that both brain regions are involved in motor learning and motor control, sensory perception integration, and reward [41–43]. The cerebellum sends projections to the SN, regulating the release of dopamine within the basal ganglia [44]. The STN of the basal ganglia also sends disynaptic projections to the cerebellar cortex [45]. These connections allow the cerebellum to provide feedback on information received from the basal ganglia, “fine tuning” the basal ganglia motor and reward processes. Thus, in dystonias that result from impaired PC function, dystonic symptoms could arise from aberrant cerebellar output to the basal ganglia. Indeed, abnormal cerebellar activity can modulate cortical excitability and basal ganglia function, exacerbating motor symptoms [46].

The few therapeutic interventions for dystonia that target the basal ganglia focus on increasing the release of dopamine to alleviate motor symptoms. DYT5-GCHI (“Dopa-responsive dystonia”) patients exhibit a decrease in dopamine in the SN, and many respond positively to levodopa (L-DOPA) treatment [47]. Parkinson’s disease, which also includes a decrease in the dopamine production of the SN, includes dystonia as a symptom, and patients also responds positively to L-DOPA treatment. Therefore, targeting the cerebellum could be a more viable and efficacious therapeutic option for patients with basal ganglia neuronal dysfunction.

Human studies have shown burst firing of neurons in the globus pallidus (GP) of the basal ganglia within dystonia patients [48]. Parkinson’s disease and patients with combined or complex dystonia have also displayed increased bursting and oscillatory activity in the STN, as well as abnormal cerebellar activity [49–51]. Burst firing in the STN could result in increased cerebellar activity in both disorders and alter the information sent to the motor cortex in the cerebellothalamocortical loop. Deep Brain Stimulation (DBS), which involves the surgical implantation of an electrode that administers a controlled electrical current, in the cerebellum has emerged as a new therapeutic treatment for dystonias with a basal ganglia etiology [52, 53]. Thus, it is important to view the basal ganglia and cerebellum as a bi-directional network in the context of impairment in dystonia.

## Cerebellar circuit

The cerebellum, latin for “little brain,” is located at the back portion of the brain directly underneath the cerebral cortex and just above the spinal cord in humans. Traditionally, the cerebellum is known for its involvement in motor coordination, motor learning, and eye control [34, 35, 54]. More recently, the cerebellum has gained attention for its non-motor contributions towards emotions and cognition [55, 56]. While cerebellar dysfunction is known to contribute to several movement disorders such as dystonia, ataxia, and Parkinson’s disease, cerebellar dysfunction has also been linked to non-motor disorders such as autism spectrum disorder, obsessive-compulsive disorder, and schizophrenia [56]. Here, we will focus on how cerebellum dysfunction contributes to different dystonias through aberrations of neuronal subtypes such as PCs and CN. However, first we provide a brief overview of the local cerebellar circuit.

**Figure 2.**
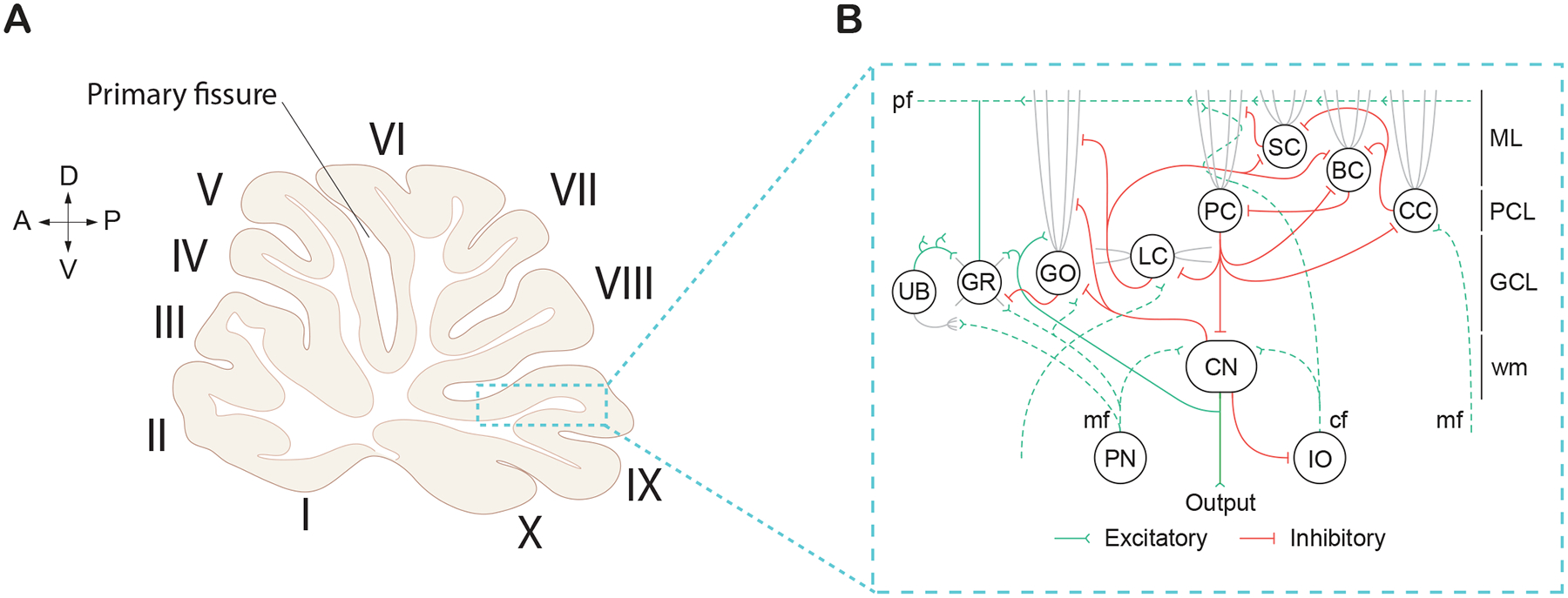
Cerebellar cortex morphology and cellular connections in a rat brain. **(A)** Sagittal midline cerebellar section displaying the cerebellar lobules (I-X) in Roman numerals. Arrows displayed for orientation of anterior (A), posterior (P), dorsal (D), and ventral (V) directions. Adapted from (57) **(B)** Circuitry of the cerebellar cortex, which centers around Purkinje cells (PC), which are the sole output of the cerebellar cortex. Cell types also include interneurons: Unipolar brush cell (UB); Golgi cell (GO); Lugaro (LC); Stellate cell (SC); Basket cell (BC); and Candelabrum cell (CC), and excitatory Granule cells (GR) which send excitatory inputs onto PC’s via parallel fibers (pf). PC’s send inhibitory projections to the cerebellar nuclei (CN), and receive excitatory inputs from the Pontine nuclei (PN) and the Inferior olive (IO) via the mossy fiber (mf); climbing fiber (cf) inputs respectively. The cerebellar cortex is separated into multiple layers, including the Molecular layer (ML), Purkinje cell layer (PCL), Granule cell layer (GCL), and white matter (wm). Schematic adapted from (211).

On a macroscopic scale, the cerebellum is divided into two hemispheres by the vermis [57–61]. These hemispheres are further subdivided into three distinct lobes: a small anterior lobe, a large posterior lobe, and a tiny flocculonodular lobe. These lobes are produced as the result of two deep fissures within the cerebellum. The anterior and posterior lobes are separated by the primary fissure, while the posterior and flocculonodular are separated by the posterolateral fissure [57, 58]. Finally, the lobes are subdivided into lobules (I-X) by shallow fissures (Figure 2A). Lobules I-V are part of the anterior lobe, lobules VI-IX are part of the posterior lobe, and lobule X is part of the flocculonodular lobe.

On a cellular level, the cerebellar cortex is divided into three distinct layers (Figure 2B). Each layer contains at least one of the major cerebellar neuronal subtypes: PC, basket cells (BCs), stellate cells (SCs), granule cells (GRs), Golgi cells (GOs), Lugaro cells (LCs), and/or unipolar brush cells (UBCs; [57, 59, 60, 63]. The molecular layer is the outermost layer situated right beneath the cerebellar surface. The molecular layer contains basket and stellate cells, as well as the parallel fibers (pf) from the granule cells (GC), climbing fibers (cf) from the inferior olive (IO), and dendrites from the PCs (Figure 2B). Below the molecular layer is the PC layer, which contains the soma of PCs arranged in a monolayer. Situated underneath the PC layer is the granule cell layer which contains the granule cell bodies, Golgi cells, Lugaro cells, unipolar brush cells, and mossy fibers (mf) which originate from extracerebellar regions (Figure 2B). Lastly, is the white matter which contains the CN.

Despite subtle differences in cell size, density, and molecular composition between regions within the cerebellum, the cerebellar circuit is highly organized and regular [57, 59, 64]. The cerebellum receives extracerebellar information from mossy fibers which form synaptic junctions onto excitatory granule cells and inhibitory Golgi cells. The cerebellum also receives information from the brainstem through the inferior olive which projects climbing fibers excitatory synapses onto the dendrites of PCs. While there are two major sources of inputs to the cerebellar cortex, PCs are the only neuronal output of the cerebellar cortex. This means that the output from PCs onto CN reflects all the computations performed in the cerebellar cortex [59, 63, 65].

PCs are large, GABAergic inhibitory neurons important for relaying information out of the cerebellar cortex [59, 63]. Despite the small number of PCs (~15 million) compared to other neuronal subtypes such as granule cells (10–100 billion), PCs have become rather synonymous with the cerebellar cortex [58, 66]. PCs are responsible for integrating excitatory signals directly from the climbing fibers by means of the inferior olive. PCs also integrate signals indirectly from mossy fibers. Additionally, PCs receive inhibitory signals directly from basket and stellate cells [58, 59]. The axon of the PCs projects towards the white matter to form inhibitory synapses with CN [57, 58]. PCs are unique in that they are capable of two forms of action potentials, both simple spikes (20–200 Hz) and complex spikes (~1 Hz; [57, 65, 67]. Simple spikes are generated by both PC intrinsic pacemaking activity [63, 68] and parallel fiber activity [58, 69]. Whereas complex spikes, which appear as a large action potential followed by a burst of attenuated spikelets, are generated by climbing fiber activity [57, 58, 63, 65, 69].

Both synaptic and intrinsic PC processes are highly dependent on Ca^2+^ dynamics. In fact, PCs have some of the highest levels of endogenous Ca^2+^ buffering capacity due to their expression of Ca^2+^ binding proteins such as parvalbumin (PV) and calbindin D-28K (CB) [70, 71]. Voltage-gated Ca^2+^ channels, such as Cav2.1 (P/Q type) channels discussed later in this review, play an essential role in intrinsic activity through its coupling with large and small Ca^2+^-activated potassium channels (BK and SK channels respectively) [72]. During action potentials, BK channels facilitate rapid repolarization, limiting the duration of the spike and enabling high-frequency firing [20]. SK channels are responsible for the after-hyperpolarization, which maintains PC intrinsic firing [73]. Furthermore, Ca^2+^ release from intracellular stores, mediated by ryanodine and inositol 1,4,5-triphosphate receptor (IP_3_R1) receptors, contributes to dendritic Ca^2+^ spikes, which integrate synaptic inputs and generate complex spike activity [74].

How these inputs control PC activity is also dictated by Ca^2+^ dynamics, because Ca^2+^ influx through Cav2.1 channels and AMPA receptors is essential for synaptic PC plasticity mechanisms such as long-term depression (LTD) and long-term potentiation (LTP). LTD, induced by the coincident activation of parallel and climbing fiber inputs onto PCs, depends on a Ca^2+^-dependent signaling cascade involving protein kinase C (PKC) and the internalization of AMPA receptors, resulting in decreased synaptic strength [75, 76]. In contrast, LTP involves Ca^2+^-dependent pathways that enhance AMPA receptor function, strengthening synaptic transmission [77]. These complementary plasticity mechanisms allow PCs to modulate cerebellar output adaptively, refining motor commands and supporting motor learning [78].

PCs then send this information through monosynaptic inhibitory projections to the CN in the white matter of the cerebellum (Figure 2B; [63, 78]). CN typically receive inputs from ~40 converging PCs [79]. PC modulation of CN are highly influenced by synaptic interactions with climbing fibers and mossy fibers result (Figure 2B) [80, 81]. There has been some debate in the field as to whether PCs modulate CN activity by synchronizing their firing patterns, also referred to as a “temporal code,” or through changes in their firing rate, or “rate code.” In the temporal code, synchrony has been thought to be essential for controlling the timing and precision of movement, as the CN relay motor signals to various brain regions. Studies have shown that PC firing patterns shape the output of the CN, with disruption of this synchrony leading to motor deficits such as dystonia [82]. Additionally, the CN themselves are involved in generating rhythmic activity that is finely tuned by PC inputs, contributing to motor execution and coordination [83, 84]. Rate coding suggests that the firing rate of PC encodes information, with higher firing rates representing stronger signals or more significant neural events [85]. There is a vast amount of evidence for a rate code for behaviors where PC and CN firing rates are inversely correlated, increased PC activity results in decreased CN activity [86–88]. While the verdict is still out, recent work suggests both temporal and rate code could be contributing to CN output depending on the variability of the input and synapse size [89].

These nuclei serve as a relay station to carry information out of the cerebellum to extracerebellar regions like the thalamus and brainstem [60, 90]. The CN can be divided into four main subgroups: the dentate nucleus, the interposed nucleus which is subdivided into the emboliform (anterior) and globose (posterior) nucleus in humans, and the fastigial nucleus [58, 60, 90]. Each of these subgroups receives efferent signal transduction from PCs in different regions. The dentate nucleus receives information from PCs in the cerebellar hemispheres while the fastigial nuclei receive information from PCs in the vermis. Additionally, the interposed nucleus receives information from PCs in the paravermis [60, 90, 91]. The CN then make monosynaptic connections to many extracerebellar regions including, but not limited to the locus coeruleus, red nucleus, thalamus, substantia nigra, and hypothalamus. However, there are regions like the cerebellar cortex and the basal ganglia that receive disynaptic input from the cerebellum via the thalamus (Figure 1; [90, 92]). The CN projections from the cerebellar cortex can either be glutamatergic, GABAergic, or glycinergic in nature [93–95]. Similar to PCs, the CN have intrinsically active pacemaking activity [96, 97]. Moreover, dysfunction of the CN has been implicated in several models of dystonia [15, 98, 99]. Given the importance of PCs in relaying information out of the cerebellar cortex through the CN, it is understandable how the dysfunction of PCs and CN contribute to neurological disorders, such as dystonia.

## Dystonia and the cerebellar cell types

In humans, understanding the neuropathology of dystonia is limited due to relatively small sample sizes of previous characterization studies. This is particularly relevant when attempting to elucidate mechanistic contributions of the cerebellum to disease pathogenesis. However, neuroimaging studies from human samples can provide preliminary insights that the cerebellum, as well as the basal ganglia, contribute to several forms of dystonia [100–103]. Several neuroimaging techniques are used to study and identify how abnormalities in the cerebellum correlate with different forms of dystonia. These techniques include functional magnetic resonance imaging (fMRI), fluorodeoxyglucose positron emission tomography (FDG-PET), PET blood flow studies, diffusion tensor imaging studies (DTI), and voxel-based morphometry (VBM). Additionally, immunostaining of post-mortem human brains can provide additional information towards understanding the mechanistic causes for the pathogenesis of dystonia. Each of these techniques come with its own benefits and limitations; however, individually, these techniques fail to identify the cellular and molecular neuropathology associated with dystonias. Therefore, it is necessary to combine animal models with known human data sets to characterize the neuropathology of dystonia. To date, information regarding human and animal models is consolidated based on groupings isolated by individual or combined dystonia phenotypes. In this review, we highlight human studies and animal models with dystonia phenotypes that exhibit dysfunction of PCs and/or CN (Table 2).

### Isolated dystonia subtypes

#### DYT-TOR1A (previously DYT1)

DYT-TOR1A, previously known as DYT1, is a form of dystonia with an autosomal dominant inheritance that leads to early-onset generalized dystonia. The onset of DYT-TOR1A starts in childhood (mean = 13 years; range = 1–28 years) [24, 25]. Typically, disorder-associated phenotypes begin in one limb and progressively become generalized over time [25, 130]. Patients diagnosed with DYT-TOR1A generally display uncoordinated and abnormal movements that are particularly evident during action-specific movements like walking or fine motor movements like writing [131, 132]. Additionally, patients experience muscle tone abnormalities including stiffness and spasms that results in rigid inflexible limbs. While symptoms can be as minor as a writer’s cramp, the dystonic postures in the limbs often lead to disruptions in gait and writing [133, 134].

The underlying cause of DYT-TOR1A is a 3-base pair GAG deletion in the TOR1A gene, which encodes TorsinA. This GAG deletion ultimately leads to the removal of glutamic acid from the c-terminal portion of the protein [135]. TorsinA is a member of the AAA+ ATPase family, and this protein is involved in various cellular activities such as maintaining cell polarity in migrating cells, homeostasis against ER stress, trafficking membrane proteins, and protein secretion [25, 130]. In humans, TorsinA is found in many brain regions including the cerebellum. Moreover, TorsinA is highly expressed in PCs and neurons of the dentate nucleus during early developmental stages [136, 137]. The highest expression of TorsinA has been found at the dendritic spines and axon terminals of PCs [138].

Multiple human neuroimaging studies have identified metabolic and network abnormalities in the cerebellum of DYT-TOR1A patients. FDG-PET studies show increased uptake of tracer in the cerebellar hemisphere which is normally associated with increased metabolic activity induced by inflammation, infection, or malignancy [100–102]. Similarly, several groups using PET scans have shown increased cerebral blood flow in the cerebellum of DYT-TOR1A patients [102, 104, 139, 140]. This is indicative of metabolic abnormalities associated with disease phenotypes. Finally, DTI tractography has shown decreased connectivity between the cerebellum and the thalamus [11, 102]. However, knowing there are broad alterations in the cerebellum is not enough. To truly understand the neuropathology of DYT-TOR1A we must understand the changes that occur at the cellular level. This is currently being undertaken using various animal models.

Of the different subtypes of dystonia examined using rodent models, DYT-TOR1A is perhaps the most characterized. Multiple DYT-TOR1A animal models have been created in which the TorsinA protein is globally or regionally/cell-type specifically knocked-down KD; [141], knocked-out (KO; [81, 82]) or the GAG mutation is knocked-in (KI; [83, 84]). Additionally, these KOs can be complete or conditional. Many DYT-TOR1A rodent models fail to show overt dystonia [114, 142–145]. This is likely due to the rodent’s ability to compensate for the loss of genes, which, makes understanding human pathology in rodent models challenging. Despite lack of an overt dystonia phenotype, many of these models contribute to the fundamental understanding of the biological role of TorsinA. The behavioral phenotypes and extracerebellar neuropathology has been reviewed previously so will only be discussed briefly [108, 146, 147]. This review focuses on consolidating the knowledge pertaining to PCs and CN within the context of different dystonia subtypes.

The DYT-TOR1A ΔGAG allele KI model is the most similar to human dystonia patients in that only one of the pair of glutamic acid residues in the TorsinA protein is removed [108]. The DYT-TOR1A ΔGAG KI mouse model exhibits dystonia-like motor deficits that differs depending on the behavioral protocol utilized. DYT-TOR1A ΔGAG KI mice show deficits in motor coordination and balance on the beam-walking assay, increased activity levels in the open field test, mild abnormality in gait, and no deficits in motor learning on the rotarod [142, 148]. These changes are not associated with typical dystonic postures which includes alterations in hindlimb clasp, truncal posture, or righting reflexes [142]. In separate studies examining the neuropathology of DYT- TOR1A ΔGAG KI mice, the cerebellum appears well developed with normal expression and density of PCs, granule cells, and CN [105, 108, 142]. While macroscopic changes in the cerebellum may not be readily observable, the cerebellum of DYT-TOR1A ΔGAG KI mice is slightly larger (~5%) than WT mice [105]. Moreover, there are subtle changes in the morphology of PCs in the DYT-TOR1A ΔGAG KI model. The PCs from mutant mice have shorter primary dendrites and reduced spine numbers on distal dendrites [19, 105, 108]. In addition to morphological changes, functional changes in PCs of DYT-TOR1A ΔGAG KI mice have been observed. In slice preparation examining PC intrinsic activity, there was no change in the electrophysiological properties of tonically firing PCs. However, in non-tonically firing PCs, there was an abnormal increase in firing rates along with a decrease in peak frequency attributed to an increase in BK channel activity. Ultimately, this did not result in an overall change in mean firing rates of the cells [17]. Similar to what was observed in humans, PET and DTI imaging of this mouse model suggests a reduction in connections between the cerebellum, thalamus, and cerebral cortex [12].

There are several regional or cell-specific DYT-TOR1A conditional KO or KI animal models to understand how regions and cell-types contribute to the neuropathology associated with dystonia. TorsinA has been conditionally knocked-out of the central nervous system [149], cerebral cortex [150], striatum [151], cholinergic neurons [152], and PCs [19, 20]. Additionally, the ΔGAG mutation has been specifically knocked-in to dopaminergic neurons and PCs [106]. Similar to DYT-TOR1A ΔGAG KI mice, the cerebral cortex-specific KO mice show motor coordination and balance deficits in beam-walking, increased locomotion in the open field test, and mild alterations in gait [150]. In the striatum-specific KO model, there were no observable dystonic postures, changes in spontaneous locomotion or gross motor skills [151]. However, motor coordination and balance deficits were noted on the beam-walking test. In the cholinergic-specific KO, there were no observable changes in spontaneous locomotion or fine motor coordination and balance skills, however, a deficit was observed in gross motor skills by a reduced latency to fall on the rotarod test [152]. In the PC-specific KO, there were no observable dystonic postures, as well as no changes in gait or gross locomotor activity on the rotarod [18]. However, improved motor coordination and balance were noted on the beam-walking test. Crossing the PC-specific KO to the DYT-TOR1A ΔGAG KI mice rescued the beam-walking deficits observed in the DYT- TOR1A ΔGAG KI model [18]. When the ΔGAG mutation was specifically knocked-in to the D2 dopamine receptors, a deficit in motor balance and coordination was observed in the beam-walking test, but not the rotarod [106]. In contrast, when the ΔGAG mutation was knocked-in to PCs, female mice showed improved motor skills on the rotarod, with no changes in the beam-walking test [106]. Like the DYT-TOR1A ΔGAG KI model, the PC-specific DYT-TOR1A ΔGAG KO model demonstrates PC morphological changes. In this model, the primary dendrites are shorter and there is a reduction in spine numbers on distal dendrites [19]. While most of these models provide valuable information about how other brain regions contribute to dystonia, there is limited information about how extracerebellar regional knock-out impacts the different cells of the cerebellum.

A third type of DYT1-TOR1A genetic manipulation includes transgenic models in which mice overexpress the mutant human TorsinA protein (hMT). Like other KI and KO animal models, these transgenic mice do not display overt dystonic behavior, but they do exhibit motor deficits [108, 109]. With hMT mice, there is conflicting results on overall activity levels where one group did not observe changes in overall activity levels in the open-field test [153], but a second group noticed an increase in activity levels [154]. Similarly, depending on the protocols there were differences in motor behavior where one group observed hMT mice had motor and balance deficits on the beam-walking test [109], a second saw poor rotarod performance [154] and a third noted slower motor learning rate on the rotarod [153]. In some instances, the hMT mice also exhibited subtle changes in gait characterized by longer stride [153] or a wider hind-base width [109]. While dopamine dysfunction is well characterized in these mice, much less has been reported on the effect of the mutation in the cerebellum [109]. One study found hMT mice exhibit alterations in synaptogenesis in PCs that received reduced inhibitory input from parallel fibers and had increased number of excitatory synapses from the climbing fibers [110]. Another study found that expression of the hMT TorsinA protein in PCs led to an increase in cytochrome oxidase, indicative of increased metabolic activity [14]. This increase was associated with an increase in the metabolism of the inferior olive. Taken together, this suggests that an increase in inferior olive activity contributes to alterations in the firing-rate properties of PCs.

To circumvent the issue of compensation and create an overtly dystonic rodent model, one group opted to regionally knock-down TorsinA in adult mice. DYT-TOR1A KD in the adult cerebellum did not result in changes in overall activity levels in the open-field test but resulted in increased dystonia scores that were characterized by abnormal hind-limb postures [98]. These abnormal postures were confirmed to be the result of abnormal muscle contractions. On a functional level, DYT-TOR1A KD in the adult cerebellum results in increases in bursting activity as well as irregular firing of PCs in awake head-restrained mice [98]. However, there was no change in the average firing rate of PCs in these animals. The increase in bursting activity and irregularity was also observed in the CN. However, these changes were associated with a decrease in the average firing rate of the CN. When evaluating the effects of TorsinA loss in a slice preparation perfused with synaptic activity blockers to examine pacemaking activity, both PCs and CN still displayed altered firing, suggesting changes in intrinsic activity [98].

While more research is necessary to fully understand the role of TorsinA in PC and CN function and development, the data from DYT1-TOR1A mouse models suggests that TorsinA is an important protein for maintaining PC dendritic morphology and electrophysiological properties. There are many other isolated and combined forms of dystonia, and while the cerebellum has been implicated in many of these, the role of PCs and/or CN has not been as extensively characterized, particularly when examining how neuronal function contributes to motor behavior.

#### DYT-THAP1 (previously DYT6)

DYT-THAP1 is an isolated form of dystonia with adolescent-onset of symptoms. It was previously referred to as DYT6 and is also known as adolescent-onset dystonia of mixed type. This subtype of dystonia was first identified in a Mennonite family. The onset of this subtype usually occurs in adolescence with features of focal dystonia beginning in the cervical or cranial muscles. Over time these features become more generalized [25, 155]. The clinical features of DYT-THAP1 can be quite similar to those of DYT-TOR1A. Since DYT-THAP1 is more likely to involve cranial and cervical muscles patients experience dysarthria and dysphagia [156–158]. Some manifesting and non-manifesting DYT-THAP1 carriers also develop a tremor that is thought to occur from cerebellar dysfunction [21, 159].

The molecular cause of DYT-THAP1 is either a nonsense, missense, or truncation mutation in the gene associated with the Thanatos-associated domain-containing apoptosis-associated protein (THAP1). Loss-of-function mutations in Thap1 have been shown to result in changes in voltage gated Ca^2+^ channel expression associated with other forms of cerebellar dystonia [160]. Human subjects, neuroimaging and tractography studies have demonstrated there is reduced connectivity between the cerebellum, thalamus, and cerebral cortex [11, 13, 102]; however, to date, not much is known about the functional roles of PCs or CN in this subtype. Studies using heterozygous KO mice found that both PC and CN cell numbers were reduced [111, 161]. In this model, PCs displayed a significant reduction in the regularity of simple spike activity without significant alterations in the firing frequency [111]. Whereas the interposed cerebellar nuclei had a significantly lower firing frequency [111]. These mice, however, did not display dystonic postures or deficits in motor behavior. Instead, these mice displayed an abnormal gait and tremor, which could be account for by lack of PC and/or CN bursting [111, 161]. However, deficits in gait and motor performance on the beam walking assay were noted in a THAP1-C54Y heterozygous mouse model [161]. This suggests that the type of genetic mutation can explain differences in motor phenotypes observed and should be carefully selected and examined.

### Combined dystonia subtypes

#### DYT-TAF1 (previously DYT3)

DYT-TAF1 is a form of combined dystonia where Parkinsonism is the predominant disorder [3]. It was previously referred to as DYT3, and is also known as Lubag, and X-linked dystonia-parkinsonism (XDP). The onset of DYT-TAF1 usually occurs in early adulthood, typically in men, between the ages of 30–45. The onset begins focally, yet eventually will spread to multiple body regions over time [103, 114]. In many cases, more Parkinson’s related features like rigidity, bradykinesia, postural instability, resting tremor and dysphagia develop with the onset of dystonia or at a later-stage of disease progression [103, 162].

The underlying cause of DYT-TAF1 is a repeat expansion in the TATA box-binding protein associated factor 1 (TAF1) gene, which is an essential part of the transcription machinery in neurons. While the neuropathology of DYT-TAF1 is primarily associated with dysfunction in the striatum [103, 163], PC loss has been noted from the postmortem tissue of human patients with DYT-TAF1 [103]. Using CRISPR/Cas9 to delete TAF1 from early postnatal rats results in both behavioral and morphological changes. TAF1-edited rat pups show a variety of motor deficits and hind-limb weakness as measured by an increased righting reflex time, a decrease in hind-limb suspension, and reduced levels of locomotion [112]. Deficits in motor behavior of TAF1-edited pups continued into juvenile stages where increased levels of locomotor activity were observed in the open field test and motor coordination and balance was decreased in the beam-walking assay. In correlation with these behavioral changes, morphological changes showed an abnormal an PC layer with reduced number of PCs, as well as a decreased thickness in the granule cell layer [112]. Functionally, the PCs of the TAF1-edited rats showed a decrease in the frequency of spontaneous excitatory postsynaptic currents (sEPSCs) which may underlie the behavioral deficits seen. Until recently, a genetic rodent model had not been established to examine the function of DYT-TAF1 presumably due to its embryonic lethality in male mice [164]. However, the heterozygous TAF1 female mice survive and can be used to study behavior. Initial behavioral testing shows that the heterozygous female mice have reduced locomotor activity in the open field test, but no changes in balance and coordination on the rotarod [164]. More research needs to be conducted with these newer animal models to examine the contribution of PCs and CN in DYT-TAF1.

#### DYT-SGCE (previously DYT11)

DYT-SGCE is a combined dystonia in which myoclonus is the prevalent disorder.

This subtype has previously been classified as DYT11 and is also known as myoclonus-dystonia (MD). The onset of this dystonia subtype is usually between childhood and adolescence [25]. The predominant motor phenotype for patients is the myoclonic jerk, which is a brief and sudden muscle contraction that normally effects the head, trunk, and upper limbs. Roughly half of these patients present with a form of focal or segmental dystonia such as writer’s cramp or spasmodic torticollis [165, 166]. DYT-SGCE is also characterized by slower saccadic adaptation to a visual target, which is thought to arise from dysfunction in the cerebellum [167].

The underlying cause of DYT-SGCE is deletions of part of the ε-sarcoglycan gene (SGCE) that results in gene loss of function. Worth noting, SGCE is highly expressed in the cerebellum, particularly within the PCs and the dentate nuclei, compared to other brain regions implicated in dystonia [168]. In humans, the cerebellum has been implicated in DYT-SGCE from FDG-PET scan findings that observed increased metabolism within the cerebellum [113]. Additionally, exome sequencing revealed a mutation in the CACNA1B gene, which encodes the voltage gated Ca^2+^ channel Cav2.2 as the causal gene in a family with a unique dominant myoclonus-dystonia-like syndrome [169].

Like the other animal models discussed, SGCE KO mice do not display overt dystonic postures. However, DYT-SGCE KO mice are characterized by myoclonic jerks and impaired motor performance and motor learning on the beam-walking assay [170]. Additionally, global SGCE KO mice display increased spontaneous locomotion during open field tests [170]. In contrast, the PC-specific SGCE KO model which does not show dystonic or myoclonic postures, only showed deficits in motor learning, but not motor performance in both the beam walking and rotarod assays. Morphologically, in the global SGCE KO mice, there was an abnormal nuclear envelope morphology in PC that is not conserved in PC-cell specific KO mice [107]. However, acute shRNA knock-down of SGCE in the cerebellum of the adult mouse leads to overt dystonic postures, especially in the hind limbs and tail, along with a mild ataxic gait and myoclonic jerks [99]. These motor phenotypes were correlated with abnormal firing properties of both PC and CN cells. In this study, both cell types displayed a reduction in average firing rate and an increase in firing regularity. However, PCs also show an increase in their mode firing rate in this model system [99].

#### DYT-ATP1A3 (previously DYT12)

DYT-ATP1A3 is a combined dystonia in which Parkinsonism is the prevalent disorder. This subtype has previously been known as DYT12 and is also known as Rapid-onset Dystonia Parkinsonism (RDP). With regards to the cerebellum, this is perhaps the second most studied and characterized subtype of dystonia in rodent models. The onset of DYT-ATP1A3 varies between adolescence and young adulthood [3, 25]. The primary onset of RDP typically occurs after a physiological (physical or psychological) stressor [171, 172]. Primary symptoms include bulbar dystonia which leads to dysarthria and dysphagia, as well as, dystonia in the limbs, particularly in the arms and hands. The more Parkinsonian-associated symptoms of RDP include slowness of movement, postural instability and hypophonia [171, 172].

The underlying cause of DYT-ATP1A3 is a loss-of-function missense mutation in the ATP1A3 gene. This gene encodes the α3 subunit of the sodium-potassium adenosine triphosphate pump (Na^+^/K^+^ ATPase; [173]. While the Na^+^/K^+^ pump is expressed throughout the brain, PCs exclusively express the α3 subunit [174]. While most human studies of dystonia implicate the cerebellum, postmortem analysis of DYT-ATP1A3 patients directly implicates the different cell types of the cerebellum. Postmortem staining of symptomatic RDP patients displayed a reduced number of PCs, granule cells, and neurons in the dentate nucleus. Moreover, the remaining PCs displayed an abnormal morphology consisting of axonal swelling (torpedoes) and reduced dendritic arborization [114, 115].

DYT-ATP1A3 has been studied using genetic and pharmacological animal models. While genetic models knock-down ATP1A3, pharmacological models selectively inhibit the Na^+^/K^+^ pump using ouabain [15, 16, 117]. The shRNA ATP1A3 KD model produced mice with significant dystonic postures in the limbs caused by co-contraction of agonist and antagonistic muscle groups [16]. Additionally, in correlation with the behavioral phenotype, this model exhibited both PCs and CN with abnormally high-frequency bursting patterns. This was not associated with a change in the average firing rate [16]. While this was associated with changes in the intrinsic activity of PCs, there was no change in the intrinsic activity of CN. In a heterozygous ATP1A3 KD model, mice exhibited increased spontaneous locomotor activity and enhanced motor performance on the rotarod and beam-walking assays, but no overt dystonic postures [116]. Nevertheless, an increase of inhibitory neurotransmission onto PCs from interneurons was observed in the molecular layer. However, symptoms of RDP are known to arise when exposed to a physiological stressor. When female heterozygous ATP1A3 KD mice are exposed to restraint stress, deficits in motor function occur on both the beam-walking and rotarod assays, though there is no change in grip strength [175]. Together, this suggests that changes in synaptic plasticity may contribute to symptoms of dystonia seen after a stressor [116].

Pharmacological findings align well with findings from the genetic model. In adult mice and juvenile rats cerebellar infusions of ouabain induced dystonic postures [15]. In the ouabain-induced dystonia model, PCs and CN both displayed persistent increases in burst-firing activity. Moreover, there were no changes in mean firing rate under these experimental conditions [15].

## Animal models with dystonia phenotypes

The previously mentioned animal models were associated with genetic mutations that were found in humans. However, several other genetic and pharmacological rodent models display dystonic motor phenotypes that are thought to be caused by abnormal activity of PCs. Below we will discuss some rodent models and how PC activity in these models is thought to contribute to phenotypes associated with dystonia.

### Tottering mouse

The tottering mouse (tg), results from a missense mutation in the Cacna1A gene that encodes the α1 subunit of the P/Q-type voltage Ca^2+^ channel (Ca_v_2.1; [176]. This mouse model is characterized by several phenotypes including but not limited to paroxysmal dystonia [177], ataxia [118, 178], and absence seizures [179, 180]. While best known as an animal model for episodic ataxia type 2 (EA2), examination of PC abnormalities has provided information regarding the neuropathology of dystonia. Interestingly, both global and PC specific expression of the Cacna1A mutation is sufficient to induce dystonia phenotypes [181, 182]. In these mutant mice, PCs exhibit high-frequency bursting activity that is not associated with changes in the average firing rate [118–120]. Moreover, the severity of motor dysfunction was correlated with the degree of abnormal firing [118]. While a complete cerebellectomy reduces the dystonic motor phenotype in tottering mice [7], selective removal of PCs through a process of degeneration is sufficient to abolish the motor dysfunction [181].

### Leaner mouse

Like the tottering mouse, the leaner mouse (tg^la^) results from a mutation in the α1 subunit of the Cav2.1 channel. The motor deficit phenotypes of these mutants is much more pronounced than the tottering mice and include generalized dystonia and ataxia [4]. In homozygous leaner mice, both granule cells and PCs show pronounced cell degeneration. By ~6 months of age there are respectively ~45% and ~20% of cells remaining compared to WT [121]. PC death is most evident in the anterior lobe of the cerebellum and is restricted to alternating Zebrin II- bands [121, 183]. Interestingly, the severity of the dystonia phenotype has been found to decrease as the overall percentage of PC death increases [122]. Before PC death, the activity of PCs is altered. This may explain why dystonia-associated phenotypes are worse in younger animals. More specifically, in the homozygous leaner mice the intrinsic pacemaking activity of PCs is highly irregular [22]. In tandem with the increased irregularity, there is a reduction in the Ca^2+^ current density with no change in voltage-dependence of the channel [184].

### Ip3R1 mouse

The inositol 1,4,5-triphosphate receptor (IP_3_R1) homozygous and heterozygous mouse models are generally associated with spinocerebellar ataxias, seizures, and premature death in homozygotes [185–187]. However, conditional knockout of IP_3_R1 in the cerebellum and brainstem is associated with a more dystonia-like phenotype [123]. IP_3_ receptors are localized on the endoplasmic reticulum and are essential in regulating intracellular Ca^2+^ concentrations [188]. The IP_3_R1 receptor is highly expressed in PCs where it plays an essential role in maintaining PC dendritic connections with parallel fibers [189–191]. When IP_3_R1 was knocked out of the cerebellum, PCs displayed abnormal complex spike activity during dystonic behaviors. More specifically, when compared to controls there was an altered frequency of complex spikes that was lower during extension postures and higher during rigid postures [123].

### DT rat

The genetically dystonic (dt) rat is the result of a spontaneous mutation in the Atcay gene which encodes the caytaxin protein important for neurodevelopment and neurotransmission [192]. The dt rat models, severe generalized dystonia in the trunk and limbs that progressively worsens with age until death at around 40 days old [193]. Similar to other models of dystonia, metabolic studies of the dt rat report deficits in the connectivity between the cerebellum and other regions. One study found there were abnormalities between the cerebellum and the locus coeruleus, and the SN [194]. In dt rats, there are generally no reports of anatomical abnormalities in the cerebellum, PCs, or CN [195, 196]. However, at least one study has noted a subtle anatomical change in that the PC soma of dt rats was smaller with no changes were observed in the size or complexity of the dendritic arbor [197].

While dt rats may not be characterized by anatomical abnormalities there are noted neurochemical and electrophysiological abnormalities. In terms of neurochemical changes, PCs in dt rats show elevated levels of gamma-aminobutyric acid GABA; [147, 198], while the CN show a likely compensatory reduction in glutamic acid decarboxylase (GAD) activity and GABA receptor density [147, 198, 199]. Electrophysiology data from both PCs and CN display abnormal activity. PCs primarily have a reduction in the frequency and firing rates of complex spikes [124, 125]. This was correlated with reduced or unchanged simple spike firing rates respectively depending on if rats were anesthetized or awake. These deficits are thought to occur from disrupted climbing fiber input to PCs due to lack of harmaline-stimulated PC activity in dt rats [124]. Similar to PCs, in awake dt rats, the medial, interpositus, and dentate CN display rhythmically increased burst firing with no change in average firing rate [126]. However, at least one study found that the increase in rhythmicity was associated with increased firing rates, at least in the medial CN [196]. Interestingly, both a complete cerebellectomy or CN-specific lesions are sufficient methods to ameliorate the dystonia phenotype in dt rats [193, 200]. Taken together this suggests that while there is abnormal PC and CN activity, disruptions of the olivocerebellar level may play a major role in the dystonia phenotype of dt rats [124, 125].

### Glutamate receptor activation rat (or mouse)

Thus far we have discussed genetic animal models that implicate PC dysfunction in dystonia. However, there are other pharmacological rodent models of dystonia that are marked by PC dysfunction. For example, injection of kainic acid or AMPA receptor agonists into the cerebellum results in generalized dystonia associated with aberrant PC function [127–129]. When kainic acid is injected into a mouse model lacking PCs, dystonia is significantly reduced [129]. These findings are not surprising considering AMPA receptor agonists have been shown to influence cultured PC firing rate in [201].

## Discussion

Historically, dystonia has been characterized as a group of neurodegenerative disorders resulting in dysregulation of the basal ganglia motor loop. Generally, researchers now agree that dystonia results from the dysfunction of the connectivity of brain regions in the cerebello-thalamo-cortical pathway [11, 13, 102]. Some forms of dystonia respond well to DBS of the basal ganglia [52, 53], while others respond positively to DBS of the cerebellum [202, 203]. The question then arises, where does the locus of dysfunction for each dystonia reside? Many forms of dystonia covered in this review display dysfunction of the cerebellar circuitry, and recent work demonstrated the cerebellar modulation basal ganglia function [204]. This suggests in dystonia, dysfunction of the cerebellar circuitry could contribute to the worsening of basal ganglia dysfunction.

Thus, a better understanding of the dysfunction of the cerebellar circuitry in dystonia could provide molecular mechanisms to alleviate symptoms. To date, many studies have been performed to elucidate the mechanistic causes of dystonia. These studies have included *in vivo* neuroimaging of humans, post-mortem brain immunostaining, animal models, and cell culture experiments from patient derived cell lines. To study the mechanistic causes in detail, there needs to be improved models of the dystonia subtypes. Rodent models are instrumental in the recapitulation of clinical manifestations discovered within afflicted patients. Albeit a limitation of animal studies is possible compensation of genetic loss, resulting in masking of the phenotype seen in humans, as was the case with many DYT-TOR1A mouse models. However, one method to combat this developmental compensation was circumvented with viral injections knocking down TorsinA in the adult cerebellum [98]. Following sufficient knockdown, mice displayed dystonic symptoms, shedding light on the PC and CN involvement in DYT-TOR1A.

It is possible PC and CN dysfunction are a lynchpin in the pathology of many dystonias involving cerebellar dysfunction, as is the case with ataxia. The timing and pattern of PC activity are integral factors in CN activity and motor coordination [80, 81]. Desynchronized PC’s, which results from increased irregular firing, could impair the synchrony of the CN, resulting in motor impairment. Indeed, distinct CN firing patterns result in ataxia, dystonia, and tremor. Thus, ataxia and dystonia exist on a spectrum of PC and CN firing, where irregular intrinsic firing results in ataxia, while a further increase in irregularity results in dystonia [205]. Many of the disorders which result from mutations or deletions of different genes that exhibit high expression patterns in the cerebellum covered in this review display irregular PC and CN activity, as well as cerebellar morphological changes. Impaired PC output can lead to cerebellar hyperactivity and impaired modulation of downstream motor circuits. This dysregulation can alter cerebellar-basal ganglia communication, contributing to the maladaptive plasticity and motor symptoms observed in dystonia.

PCs, have high Ca^2+^ buffering, and greatly depend on Ca^2+^ dynamics for the maintenance of their intrinsic activity [206, 207]. Interestingly, many of the genes covered in this review are important in homeostatic maintenance of intracellular Ca^2+^ dynamics. SGCE is associated with Ca^2+^ ion binding [208], TorsinA has been hypothesized to interact with Ca^2+^ channels and Ca^2+^ ion-related proteins like TRPC3 [209], ATP1A3 activity results in Ca^2+^ movement across the plasma membrane [210], and impairments in the TAF-1 gene alters the expression of voltage-gated Ca^2+^ channels [112]. The impact of mutations of these genes on PC or CN calcium dynamics remains to be studied in detail, but clues can come from studies utilizing animal models with a dystonia phenotype like the *tottering* mouse model of EA2. This disorder results from loss-of-function mutations in the Cav2.1 voltage gated Ca^2+^ channel, resulting in decreased influx of Ca^2+^ and baseline ataxia. Using the *tottering* model, researchers found stress-induced attacks of dystonia in EA2 occur due to increased activity of a kinase called casein kinase 2 (CK2) [120]. This kinase phosphorylates small Ca^2+^ activated potassium (SK) channels, which are vital in the after hyperpolarization of the PC action potential and necessary for the maintenance of the intrinsic firing. Phosphorylation of SK results in decreased Ca^2+^ binding, decreased channel activation, aberrant firing of PCs and dystonic attacks [120]. Because the cerebellum sends projections to the basal ganglia, aberrant cerebellar output could further worsen any dysfunction in basal ganglia circuitry, worsening symptoms.

Studying the functional and neuronal connections of the cerebellum to other brain regions like the basal ganglia in models of dystonia will shed light on the contribution of both brain regions to dysfunction. The advent of advanced Ca^2+^ imaging and *in vivo* electrophysiological recording techniques now allow us to record from multiple cell types and brain regions simultaneously. This gives us the ability to investigate cerebellar Ca^2+^ dynamics and PC functional connectivity. Furthermore, these advances allow us to examine how dysfunction in Ca^2+^ dynamics and PC connectivity contributes to each region of the cerebellar-thalamic-cortical loop. Additionally, the development of novel faster Ca^2+^ indicators will allow the field to investigate the fast Ca^2+^ dynamics seen in PCs, which will shed light on the essential role Ca^2+^ plays in dystonias. While mesoscale Ca^2+^ imaging in multiple subcompartments of PCs will give researchers the resolution to better understand how somatic and dendritic Ca^2+^ dynamics in large populations of PCs differ within a behavioral modality [211]. With these techniques performed in rodent models of dystonias, combined with tracing and *in vivo* electrophysiology of multiple regions in this loop during motor coordination, perhaps the field can increase the understanding of the connection between these regions in health and pathological states and relate it to subcellular mechanisms such as dysregulation of Ca^2+^ dynamic in PCs, providing possible pathways in PCs that could be used as a therapeutic avenue for alleviating motor symptoms in dystonia.

## Figures and Tables

**Table 1.** Clinical and etiological axes of dystonia Summarized from (3,24)

Axis I. Clinical Features			
Age of onset	Body Distribution	Temporal Pattern	Associated Features
**Infancy** birth to 2 years	**Focal** One body region is affected	**Static** Unchanging over time	**Isolated** (Previously “Primary”) Dystonia is the only motor feature (with exception of tremor)
**Childhood** 3–12 years	**Segmental** Two or more adjacent body regions are affected	**Progressive** Worsens over time	**Combined** (Previously Dystonia Plus) Dystonia is present with other movement disorders (e.g. Parkinsonism, myoclonus)
**Adolescence** 13–20 years	**Multifocal** Two or more non-adjacent body regions are involved	**Persistent** Present throughout the day with the same intensity	**Complex** Dystonia is present with other neurologic or non-neurologic features (e.g.: Wilson’s disease)
**Early Adulthood** 21–40 years	**Generalized +/− leg involvement** The trunk and at least two other regions are involved. Is further distinguished with or without leg movement	**Paroxysmal** Typically induced by a trigger (e.g. stress, caffeine)	
**Adulthood** >40 years	**Hemidystonia** Multiple body regions restricted to one side of the body	**Diurnal** Presence and severity follow a circadian rhythm	
		**Action-specific** Present during the execution of a specific task	
Axis II. Etiological Features			
Idiopathic (“Unknown”)	Genetic origin (“Inherited”)	Environmental	
		Nervous System Pathology	Acquired
**Sporadic** Isolated dystonias of unknown cause	**Autosomal dominant** Single copy of a mutated gene from one parent can lead to a genetic condition	**Degeneration** Progressive structural abnormalities such as neuronal loss	**Brain injury** During the perinatal period or due to head traumas, brain surgery or electrical injury
**Familial** Dystonia with a genetic contribution occurring with a novel gene (these often become reclassified as inherited)	**Autosomal recessive** Both parents pass on a copy of a mutated gene leading to a genetic condition	**Structural lesions** Non-progressive neuronal abnormalities or acquired lesions	**Infection/Inflammation** For example, viral encephalitis, human immunodeficiency virus (HIV) infection, autoimmune conditions, other
	**X-linked recessive** Mutation in the gene on the X-chromosome leads to a genetic condition	**No degeneration or structural lesions**	**Drugs** Levodopa, dopamine agonists, anticonvulsants and calcium channel blockers
	**Mitochondrial** Mutation in mitochondrial genes is passed from mother to their children		**Toxic** Manganese, cobalt, cyanide, methanol
			**Vascular injury** Ischemia, hemorrhage, aneurysm
			**Neoplastic** Brain tumor and paraneoplastic encephalitis
			**Psychogenic** Due to a psychological cause rather than physical one

**Table 2.** Summary of abnormal cerebellar function in dystonia animal models and animal models with dystonia phenotype

	Model	Genetic or Pharmacological Manipulation	Purkinje Cells	Cerebellar Nuclei	Other
**Isolated Dystonia**	**DYT-TOR1A humans** (100,101,113)	GAG deletion	--	--	↑ cerebellar metabolic activity ↓ connectivity between cerebellum and thalamus
	**DYT-TOR1A mouse** (17,19,118)	Δ GAG Knock-in	- shorter primary dendrites ↓ spine numbers on distal dendrites ↑ firing rate on non-tonically firing cells ↓ peak frequency in non-tonically firing cells	--	↑ in cerebellum size ↓ connectivity between cerebellum, thalamus and cerebral cortex
	**DYT-TOR1A mouse** (19,130,154)	Purkinje cell-specific knock out	- shorter primary dendrites ↓ spine numbers on distal dendrites	--	--
	**DYT-TOR1A mouse** (124,131,134)	Human TorsinA protein	↓ inhibitory input from parallel fibers ↑ excitatory input from climbing fibers ↑ metabolic activity	--	--
	**DYT-THAP1 humans** (11,13,101)	Nonsense, missense or truncating mutation	--	--	↓ connectivity between cerebellum, thalamus and cerebral cortex
	**DYT-THAP1 mouse** (141)	Heterozygous knock-out	↓ cell numbers ↓ regularity of simple spikes	↓ cell numbers ↓ firing frequency	--
**Combined Dystonias**	**DYT-TAF1 humans** (102)	Repeat expansion	- PC loss	--	--
	**DYT-TAF1 mouse** (145)	CRSPR/Cas9 deletion	- Abnormal PC layer - PC loss ↓ in PC sEPSC	--	↓ thickness in granule layer
	**DYT-SGCE humans** (151)	Deletion of part of gene	--	--	↑ metabolism in cerebellum
	**DYT-SGCE mouse** (154)	Global knock-out	- Abnormal PC nuclear envelope	--	--
	**DYT-SGCE mouse** (98)	Acute shRNA knock-down	↓ average firing rate ↑ firing regularity ↑ mode firing rate	↓ average firing rate ↑ firing regularity	--
	**DYT-ATP1A3 humans** (122,159)	Missense mutation	↓ numbers of PC - PCs with abnormal swelling and ↓ dendritic arborization	↓ numbers of neurons in the dentate nucleus	↓ numbers of granule cells
	**DYT-ATP1A3 mouse** (16)	Knockdown	↑ high-frequency burst pattern Changes in intrinsic activity	↑ frequency burst pattern	--
	**DYT-ATP1A3 mouse** (161)	Heterozygous knockdown	↑ inhibitory neurotransmission onto PC from interneurons in molecular layer	--	--
	**DYT-ATP1A3 mouse** (15,160)	Ouabain infusion	↑ burst-firing activity	↑ burst-firing activity	--
**Animal Models with Dystonia Phenotype**	**Tottering Mouse** (166,171,172)	Missense mutation in CACNA1A gene	↑ high-frequency bursting activity	--	--
	**Leaner Mouse** (22,173,175)	Mutation in CACNA1A gene	↑ PC degeneration ↑ PC death in Zebrin^−^ bands ↑ irregularity of intrinsic pacemaking ↓ Ca^2+^ current density	--	↑ granule cell degeneration
	**IP3R mouse** (180)	IP3R knockout in cerebellum	↓ frequency of complex spikes	--	--
	**DT rat** (193–195)	Mutation in Atcay gene	↓ slightly PC soma ↓ frequency and firing of complex spikes ↓ simple spike firing rate (anesthetized rats)	↑ burst firing in medial, lateral, and interpositus CN	Disrupted climbing fiber inputs
	**Glutamate Receptor Activation in rat (or mouse)** (197–199)	Kainic acid AMPAR agonists	- Dystonia symptoms are reduced in the absence of PC	--	--
